# Sometimes you’re the scooper, and sometimes you get scooped: How to turn both into something good

**DOI:** 10.1371/journal.pbio.2006843

**Published:** 2018-07-16

**Authors:** Jin-Soo Kim, Jacob E. Corn

**Affiliations:** 1 Department of Chemistry, Seoul National University, Seoul, Republic of Korea; 2 Center for Genome Engineering, Institute for Basic Science, Seoul, Republic of Korea; 3 Innovative Genomics Institute, University of California Berkeley, Berkeley, California, United States of America; 4 Department of Molecular and Cell Biology, University of California Berkeley, Berkeley, California, United States of America

## Abstract

Fast-moving, competitive fields often inadvertently duplicate research. In a research environment that values being first over being robust, this results in one manuscript “scooping” ongoing research from other groups. Opportunities to demonstrate the solidity of a result through coincidental reproduction are thus lost. Here, two group leaders, one the scooper and one the scoopee, discuss their experiences under *PLOS Biology*’s new “complementary research” policy. In this case, submission of the second article followed publication of the first by mere days. Scooper and scoopee discuss how complementary research is good for everyone by expanding the scientific reach of studies that are overlapping but not identical, demonstrating the robustness of related results, increasing readership for both authors, and making “replication” studies cost effective by creatively using resources that have already been spent.

## Introduction

We are Jacob Corn and Jin-Soo Kim, two researchers working in the fast-moving field of genome editing. What follows are our personal thoughts on experiencing PLOS’s new complementary research policy firsthand.

### Jacob Corn, “Scoopee”

It’s a nightmare most scientists have experienced at least once. On February 23, 2018, I was waiting to board a flight out of Oakland International Airport when I found out that a postdoc’s paper had been “scooped.”

Among other things, my lab works on clustered regularly interspaced short palindromic repeats (CRISPR) gene editing. Almost a year earlier, we stumbled upon the discovery that certain formats of the guide RNAs used to program Cas9 can induce a potent innate immune response. A new postdoc in the lab picked up this thread and put together a nice paper tracking down the mechanism and figuring out solutions. On February 21, 2018, the text of the paper was finished, and we were putting on the final touches. I emailed a cover letter and figures to the editor at *PLOS Biology* to gauge their interest and was gratified to get a positive response. Two days later, I was sitting in the Oakland International Airport when a beautiful paper from Jin-Soo Kim’s lab in South Korea that anticipated our own work was published [1]. There were several differences between the papers (cellular models, numbers and sequences of guide RNAs tested, etc.), but the core conclusions were similar. Dejected, I emailed *PLOS Biology* with news of the Kim lab’s paper. Like most people who have been scooped, I assumed that the work would now be relegated to languish in a drawer in my lab. That’s when the editor informed me of *PLOS Biology*’s new “complementary research” policy [2]. On February 26, 2018, we submitted our paper to PLOS Biology [3].

Many words have been written about the “reproducibility” crisis in science, and with good reason. Knowing that other groups can replicate a body of work is a critical step in establishing the strength of a scientific finding. But how to incentivize reproduction of a study? Principal investigators (PIs), postdocs, and students are all busy. Very few people want to sacrifice time and money that could be spent pursuing a new discovery in favor of replicating an older one. While my lab has personally experienced the frustration of finding that a paper doesn’t hold up, this has always been accidental rather than part of a concerted effort at reproduction.

But papers are scooped all the time. Almost everyone has experienced the pain of finding out that months or years of hard work will suddenly be a major struggle to publish, sometimes even impossible. Even more painful since the work itself has not changed in quality or importance and is only diminished in terms of precedence. Reframing very recently scooped papers as replication studies neatly solves two problems. First, results are quickly demonstrated to be robust by multiple independent labs, which is good for the scientific endeavor. Second, valuable time and resources are rescued, which is good for trainees and lab heads.

Why were scooped papers ever sent to the waste bin in the first place? Does anyone want to see them languish? Certainly not the scoopee. The scooping lab often doesn’t even know that they inflicted hardship on a colleague. The few times I’ve learned that I scooped someone, it was always inadvertent. Scooping labs actually have an incentive for similar papers to appear in print. Any reasonable study will cite the scooping paper and hopefully contribute valuable additions to strengthen the conclusions of the first study to appear. Other labs directly in the field are likely to remember who was first if two similar papers come out in rapid succession, especially if the scooped lab properly discusses and cites the first study to appear. And those outside the field often just want to know if a finding is robust, which is made easier when multiple groups have similar reports.

Of course, no one wants to incentivize “me-too” research for which a lab might jump on a bandwagon. Research is always easier once you already know the answer. The specifics of the *PLOS Biology* “complementary research” policy nicely address this. The policy favors publication of mature studies that were close to completion before being scooped and disfavors work that was in its infancy. A similar paper can only be submitted within a few months of the primary research article and only after discussion with the editor.

Publication of complementary research is quite new, so how should one manage professional relationships surrounding the work? I have no blueprints but can relate my own lab’s experience. Immediately after pressing “submit” at PLOS, I emailed Jin-Soo Kim (the lab that scooped us) to let him know that we had submitted a paper that was similar to his. I felt that it was important to let Jin-Soo know about our work so that he wasn’t caught unawares. This was an opportunity for us to have a discussion outside of the strictures of the published literature. Jin-Soo was incredibly supportive and wished us the best in our submission, for which I am immensely grateful.

In addition to making an informal connection, my group also discussed Jin-Soo’s paper in our own manuscript. We explicitly acknowledged that his work appeared first and highlighted similarities and differences between the two studies. I found this analysis interesting, since it showed that our groups had found a core result that was generally applicable. And because each paper also had its own particular set of specific experiments, the superset of the two bodies of work also strengthened each other. My group did not perform any experiments in response to the paper from Jin-Soo’s lab, since I wanted to proceed in the spirit of “accidental confirmation.” I can see arguments on both sides of this coin—either explicitly building on a recently published paper to address reproducibility of a key result or keeping each paper “clean” to avoid confusion surrounding the source of an idea. Given that my own lab was only a few days from submission, I opted to submit what we had already done.

The incredible pain of being scooped is an artificial construct. What might once have been the sting of being published second has mutated into the deep wound of not being published at all. A focus on novelty above reproducibility has led to this situation, causing problems for authors and the research community. *PLOS Biology*’s policy on complementary research is a positive step to create lasting good from a setback.

### Jin-Soo Kim, “Scooper”

As a researcher, being scooped is one of the worst things that can happen to you. Your papers or grant proposals might be rejected by journal editors or funding agencies often. In these unfortunate cases, you can always recover and resubmit them to other journals or agencies until they are accepted. But, most likely, you don’t have a second chance when you’re scooped and find yourself reading a new paper written by others that describes pretty much the same results and conclusions as yours to be written. Months or years of your efforts will have led to nowhere, a loss that can possibly ruin your career. In a competitive field of research, being scooped is inevitable and happens from time to time. I feel lucky to have survived many such cases but will never forget those moments, which still hurt.

I was very happy when I heard from Jacob Corn that his paper that had been scooped by ours was accepted for publication in *PLOS Biology* [3]. Several weeks ago, Jacob kindly informed me that he would submit a manuscript reporting on a CRISPR-RNA–mediated innate immune response in human cells. Our paper reporting the same in principle had been published in *Genome Research* earlier this year [1]. I found it very interesting and encouraging that his paper was accepted according to the journal’s new “anti-scooping” policy, which allows publication of independent but complementary or confirmatory studies. *PLOS Biology*’s new policy on complementary research will be a winner for both scoopees and scoopers. As someone who happened to be the scooper this time, I am pleased to see our findings confirmed by peers respected in the field. The publication of a second paper highlights the importance and broad interest of the findings in the original, first report. It is likely that our work will get additional attention when Jacob’s paper, in which they kindly discuss and cite our work, is published. Furthermore, the anti-scooping policy will be a winner for the scientific community because it can at least partially address the reproducibility crisis in science and enhance confidence in novel scientific findings. I enthusiastically welcome *PLOS Biology*’s new policy: it is a plus to everyone in science.
